# Clinical features of cancer with unknown primary site (clinical features, treatment, prognosis of cancer with unknown primary site)

**DOI:** 10.1186/s12885-022-10472-z

**Published:** 2022-12-31

**Authors:** HongLiang Yang, Feng He, Wen Xu, Zeng Cao

**Affiliations:** 1 Institute of Medical Engineering and Translational Medicine, Tianjin University, Tianjin, People’s Republic of China; 2 Department of Hematology, Key Laboratory of Cancer Prevention and Therapy, Tianjin Medical University Cancer Institute and Hospital, National Clinical Research Center for Cancer, Tianjin’s Clinical Research Center for Cancer, Tianjin, Ti-Yuan-Bei, Huan-Hu-Xi-Road, Tianjin, People’s Republic of China

**Keywords:** Cancer of unknown primary site, Anti-tumor, Metastasis, Clinical Features, Prognosis

## Abstract

Cancer of unknown primary site(CUPs) is a metastatic syndrome with an unidentifiable primary tumor, even after extensive workup to seek the primary site. CUPs accounts for about 3%-5% of the total number of all cancer diagnoses worldwide. The current precision medicine era has reclassified patients with CUPs into the favorable and unfavorable prognostic subset. In this study clinical characteristics and treatment of patients of CUPs were retropactively analysed. Thirty-two patients treated from July 2016 to October 2021 were included in the Affiliated Tumor Hospital of Tianjin Medical University(Tianjin, China).Common symptoms were anemia, fever, enlarged lymph nodes, abdominal pain, edema/multiple serous cavity effusion. Patients with good prognostic factors achieved good outcomes with treatment, conversely, patients with poor prognosis were generally treated empirically and had poorer outcomes. After anti-tumor treatment, the total effective rate was 41 percent(41% was the percentage of patients who achievedtumour respons). To the end of follow-up, after anti-tumor treatment, the median Overall Survival(OS) of patients was 5.4 months.

## Introduction

Cancer of unknown primary site(CUPs)is a metastatic syndrome with an unidentifiable primary tumor, even after extensive workup to seek the primary site. CUPs accounts for about 3%-5% of the total number of all cancer diagnoses worldwide [1]. The natural history of CUPs patients is completely different from that of cancer patients with a clear primary tumor. Metastasis may occurs at an early stage, with a median age at diagnosis of 65, it is slightly more common in men [1, 2] and the primary site cannot be determined clinically, the mode of metastasis is difficult to predict. In 2021 National Comprehensive Cancer Network(NCCN) guidelines for CUPs, consisted of a complete history and physical exam, laboratory tests, computerized tomographic scans, clinically directed endoscopy, and microsatellite instability(MSI)/mismatch repair gene testing. Serum tumor markers were also recommended in selected patients to seek the primary site of the tumor. The current precision medicine era has reclassified patients with CUPs into the favorable and unfavorable prognostic subset. The prognostically favorable cases account 20% of CUPs [2] and have histopathology, biomarkers, clinical presentation consistent with specific tissues of origin, may respond to standard site specific treatments, similar to primary tumors of the same site. It is important to note the subgroup of cancers with a favorable prognosis: neuroendocrine carcinomas with an unknown primary, peritoneal adenocarcinomatosis with a serous papillary subtype, isolated axillary nodal metastases in females, squamous cell carcinoma involving non-supraclavicular cervical lymph nodes, a single metastatic deposit from an unknown primary, and men with blastic bone metastases andRecently, new favorable subsets of CUP seem to emerge including colorectal, lung and renal CUP, which underlies specific treatments [3]. Latest studies using next generation sequencing(NGS) demonstrate heterogeneity among patients with both favorable and unfavorable CUPs [4]. Here we reported and summarized the clinical features of 32 cases of CUPs in Affiliated Tumor Hospital of Tianjin Medical University(Tianjin, China).

## Patients and methods

PatientS data in the Affiliated Tumor Hospital of Tianjin Medical University(Tianjin, China)were retrospectively reviewed. We enrolled 32 patients with tumors of unknown primary with complete follow-up from July 2016 to October 2021. 32 patients with metastatic malignancy were confirmed by pathological examination. All cases had complete clinical documentation and no primary lesions were found by x-ray, CT, MRI, PET-CT, endoscopy, etc.

### Data collection

Patient baseline and clinical data were collected including age, sex, laboratory tests(blood routine; blood metabolic panel: liver and kidney function, type of pathology, LDH and β_2_ microglobulin; virus detection) and imaging examination(ultrasound for superficial lymph nodes, CT or MRI for chest, abdomen and pelvic). The study was carried out in accordance with the Declaration of Helsinki and applicable local regulatory requirements and laws.

### Treatment of CUPs

#### Strong clues to the primary cites

About 20% of CUPs patients are in the good prognosis category, these CUPs have strong clues to known primary tumors and may be treated with the appropriate disease regimen [2].

- (*i*) 3 female patients with metastatic adenocarcinoma of the abdominal cavity accompanied by a marked increase in CA-125 were treated according to the stage III-IV ovarian cancer protocol(paclitaxel135mg/m^2^d1,cisplatin70mg/m^2^d2, chemotherapy cycles was 4–6).
- (*ii*) 2 male patients with metastatic adenocarcinoma of bone with elevated serum PSA were treated as metastatic prostate cancer, with anti-androgenic endocrine therapy preferred(goserelin acetate 3.6 mg subcutaneously, once every four weeks, bicalutamide 50 mg daily).
- (*iii*) 2 patients were adenocarcinomas with immunohistochemical features of colorectal cancer which could be treated with chemotherapy according to the indications for metastatic colorectal cancer(FOLFOX6: oxaliplatin 100 mg/m^2^d1, calcium folinate400mg/m^2^d1, Fluorouracil 400 mg/m^2^d1, Fluorouracil 2500 mg/m^2^d1).
- (*iv*) 6 patients with CUPs predicted to have probable bowel cancer based on 64 gene expression profiles were adopted FOLFOX6 regimen chemotherapy.

### Wreak clues to the primary cites

#### Empirical chemotherapy

The treatment of patients with poor prognosis of CUPs were based on empirical chemotherapy. In this study, carboplatin combined with epirubicin and etoposide were used(carboplatin 300 mg/m^2^d1, epirubicin60mg/m^2^d1,etoposide100mg/m^2^d1-3) for 19 patients.

### Efficacy and adverse effects

Thirty days after all treatment, the PET-CT was utilized to evaluate the treatment efficacy. Based on the NCCN 2021 guideline, the treatment response of CUPs is divided into complete remission(CR), partial remission(PR), stable disease(SD) and progressive diseases(PD). According to the WHO standard, the acute and subacute adverse cancer drug reactions were also applied to evaluate the adverse effects.

### Statistical analysis

All data were analyzed by the SPSS version 22.0 software(SPSS, Chicago, IL, United States). The survival curves were constructed by the Kaplan–Meier method. Overall survival(OS) was defined as the interval from the diagnosis of CD to death or the end of follow-up.

## Results

### Baseline characteristics

In all patients, there were 17 males(53%) and 15 females(47%). The age ranged from 19 to 76 with the median of 52 years old. The pathological type of the CUPs were predominantly metastatic adenocarcinoma(15 cases), metastatic squamous carcinoma(8 cases), neuroendocrine carcinoma(5cases) and metastatic undifferentiated carcinoma(4 cases).

### Clinical features of the patients

Common symptoms were anemia, fever, enlarged lymph nodes, abdominal pain, edema/multiple serous cavity effusion. Lymph nodes were the most common sites of the first tumor, and other sites include bone, liver, lung, peritoneum, etc. Of the 32 patients with CUPs, 16 cases were diagnosed by superficial lymph node puncture, 9 cases by focal organ biopsy, and 7 cases by finding tumor cells in pleural effusion or ascites. The basic profile of the patient is shown in Table 1.

**Table 1:** Clinical features of 32 patients

Clinical features	N(%)
Gender	
Male	17(53)
Female	15(47)
Age	
< 60	18(56)
≥ 60	14(44)
Type of pathology	
Metastatic adenocarcinoma	15(47)
Metastatic squamous carcinoma	8(25)
Neuroendocrine carcinoma	5(16)
Metastatic undifferentiated carcinoma	4(12)
Treatment options	
Treatment options with good prognosis	7(22)
Treatment options with poor prognosis	19(59)
NGS(next generation sequencing)-guided treatment	6(19)
Diagnostic site	
Superficial lymph node puncture	16(50)
Involved organ biopsy	9(28)
Malignant cells found in thoraco-peritoneal fluid	7(22)
Involved organs	
< 3	20(63)
≥ 3	12(37)
Adverse effects(grade III-IV) after treatment	
Yes	18(56)
No	14(44)

### Clinical examination

The median haemoglobin, lactate dehydrogenase, C-reactive protein were respectively 96 g/L,290U/L,28 mg/L in all patients. Carcinoembryonic antigen(CEA), CA199, NSE, squamous cell carcinoma-associated antigen(SCC), CA-125, keratin 19 fragment, and ferritin were elevated in some patients in the tumour marker assay.

### Patients treatment outcome

Thirty-two patients underwent chemotherapy treatment, the median course of chemotherapy was 4(2–6). After chemocherapy, 4 patients with complete remission(CR), 9 patients with partial remission(PR), 5 patients with stable disease(SD), 14 patients with progressive diseases(PD), and the total effective rate was 41 percent.

### Survival outcome

**Fig. 1:**
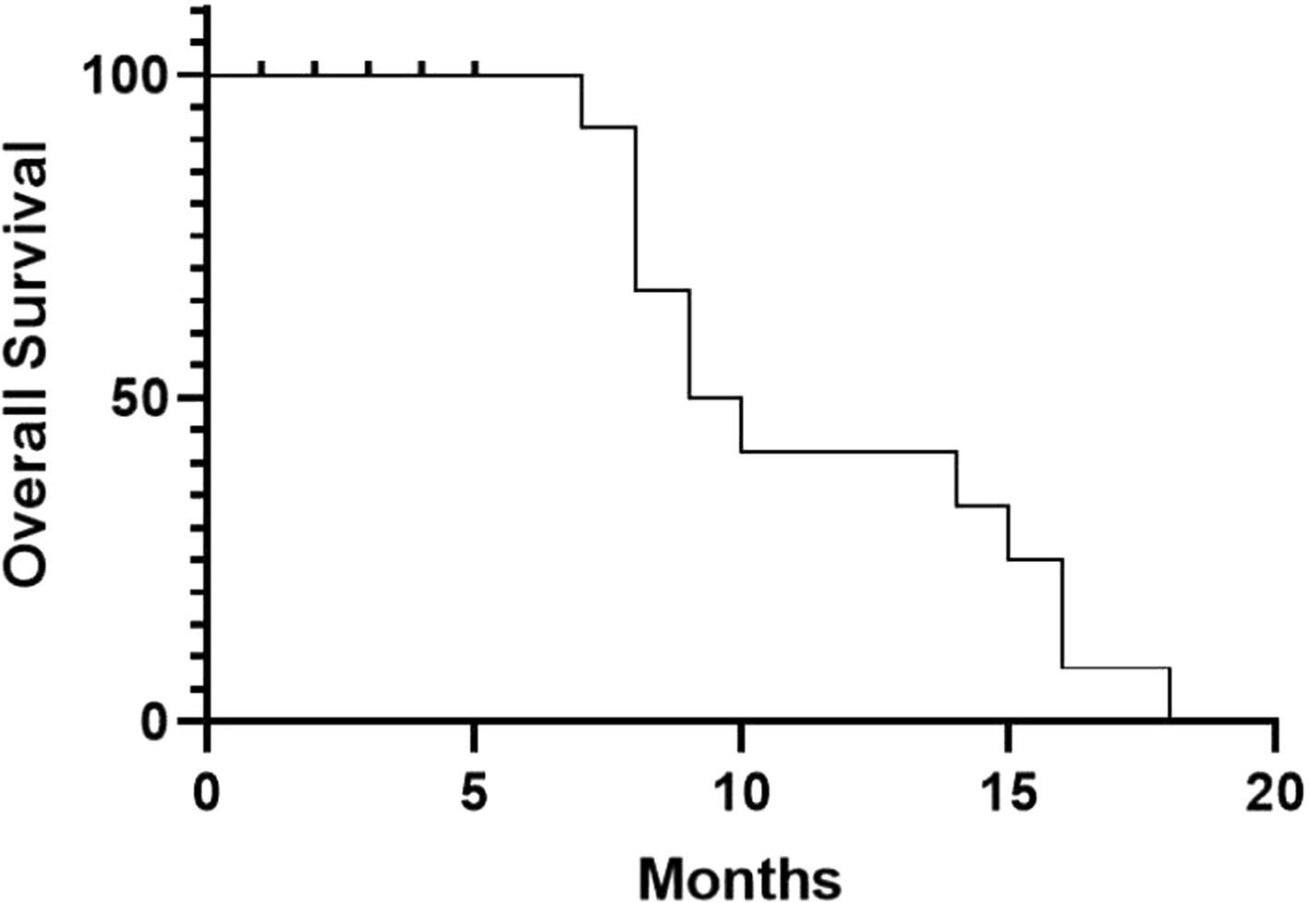
The five-year overall survival

thirty-two patients were followed up until October 2021, and no one was lost. The median follow-up months was 8 months(3–15 months). To the end of follow-up, 2 cases were diagnosed as CR, of which were metastatic adenocarcinoma. 3 patients who were metastatic adenocarcinoma were in partial remission. 4 patients got SD, one was metastatic adenocarcinoma, another was metastatic squamous carcinoma. 23 patients died of disease progression. The five-year OS of patients was 5.4 months (Fig. 1).

### Adverse effects related to the treatment

During therapy, III-IV grade myelosuppression occurred in 16 patients. After recombinant human granulocyte colony stimulating factor treatment, the leucocytes returned to normal. Adverse reactions of the digestive system, such as nausea and vomiting, occurred in 22 patients.

## Discussion

Cancers of unknown primary site(CUPs) represent a heterogeneous group of metastatic tumours for which a standardised diagnostic work-up fails to identify the site of origin at the time of diagnosis. At present, there is no international consensus on the pathogenesis of CUPs: *i* The primary lesion is recognized and attacked by the autoimmune mechanism, causing the primary lesion to recede on its own; *ii* The primary lesions are subtle and cannot be detected by the current inspection instruments; *iii* The location of the primary lesion is relatively special and cannot be reached by current examination methods; *iv* The primary tumor is not manifested at first, and the primary tumor was removed during the treatment due to drugs, radiation or surgery; *v* The course of the disease progress rapidly, and the patient die when the primary lesion is not found.

It has been reported that about 50% of CUPs patients were well-differentiated adenocarcinoma, 30% were poorly differentiated or undifferentiated adenocarcinoma, 15% were squamous cell carcinoma, and 5% were undifferentiated malignant tumor [5]. There were approximately 3% of melanomas lack an identifiable primary, otherwise known as melanoma of unknown primary(MUP). Some reports has been published that patients with MUP site seem to present better outcomes compared to those with stage-matched melanoma of known primary (MKP), probably due to higher immunogenicity as reflected in the immunologically mediated primary site regression. As such, MUP patients on immunotherapy probably display better outcomes when compared to the MKP site subset [6]. The pathological types of this study were 25%(8/32) of moderately well-differentiated adenocarcinoma, 21.9%(7/32) of poorly differentiated adenocarcinoma, 25%(8/32) of squamous cell carcinoma, and 12.5(4/32) of undifferentiated carcinoma. In our research, the proportions of squamous cell carcinoma and undifferentiated carcinoma were higher than those reported in previous literature. There are some discrepancies with the literature, which may be related to the small number of cases. This results suggests that adenocarcinoma is more common in metastatic cancers of unknown primary focus. The onset of CUPs is relatively insidious and the clinical symptoms are atypical. The first symptoms are general weakness, loss of appetite, chest tightness, abdominal distention, weight loss and unexplained lymph node enlargement which are often ignored by patients and found accidentally during physical examination or other reasons. The most common sites of involvement are lymph, liver, lung and bone, followed by pleura, peritoneum and brain. Sixteen of the thirty-two patients in this research had enlarged cervical, supraclavicular and abdominal lymph nodes as their first symptom. There were four cases of masses and vague pain in the liver area, three cases of multiple nodules in the lung tissue found by chest CT, low back pain(one case of spinal metastasis and one case of retroperitoneal metastasis), chest tightness and shortness of breath, abdominal distention(seven cases of chest and abdomen with multiple plasma cavity effusion), the origin of which could not be identified after certain clinical and pathological detailed examination.

The treatment of patients with metastatic cancer of unknown primary origin is now considered to be inextricably linked to factors such as the location of the primary tumour, the type of pathology and the stage of the tumour. Therefore, it is important to identify the primary site of the tumour as soon as possible for the patient's prognosis. CT, ultrasound, PET-CT and endoscopy should be perfected as a first step in clinical work. All of the cases in this study, the above examinations were completed, but the primary lesion site could not be found. Several gene expression profiling assays have become commercially available, claiming to blindly and correctly identify a known primary cancer and a likely tissue of origin in patients with 80% of CUPs [7, 8]. These assays are based on mRNA or miRNA RT-PCR or oligonucleotide microarray technologies [9–11]. No significant differences in the tumour microRNA expression profile were evident when CUPs metastases biologically assigned to a primary tissue of origin were compared with metastases from typical solid tumours of known origin [12]. These tests may aid in the diagnosis of the putative primary tumour site in some patients. While it is important to seek the primary lesion, treatment should not be delayed by the pursuit of examination results, and the long time and costly tests can add to the financial burden of patients. We believes that efforts should be made in the clinical diagnosis of patients with CUPs and strive to to do the following: *i* A history of previous malignancy needs to be excluded for the diagnosis of CUPs, and metastatic cancer with a clear primary focus should be ruled out by thorough history inquiry. *ii* Comprehensive physical examination: including body surface, extremities for lumps, enlarged lymph nodes, superficial body cavities such as the mouth and pharynx,nasal cavity, vagina tract, anus, etc. *iii* For new admissions, relevant tumour markers should be completed. If prostate-specific antigen(PSA) is elevated, it is often indicative of a tumour from the prostate. Neuron-specific enolase(NSE) is a marker of neuroendocrine tumours. *iv* .It is the most basic for patients to complete X-ray, B-ultrasound, CT, MRI, PET-CT and other imaging examinations. *v* Endoscopycan observe the lesion site more directlyandbiopsies can be taken to determine the nature of the lesion. *VI* Histocytological biopsy is an effective means of identifying the primary focus means.

Up to now, there is no uniform and effective treatment method for CUPs. The main treatment for CUPs is chemotherapy, which varies greatly in terms of regimen, duration, dose and route of administration. Now, more and more immunotherapy is applied to CUPs. Chromosomal instability (CIN) is not a frequent phenomenon in CUP, which may favour immune checkpoint inhibitors(ICI) among patients with CUP. Conversely, these patients present individual gene alterations implicated in immune-evasion and resistance to ICI. Further clinical investigations are needed to provide more information regarding the interplay between CIN, point mutations and the immune system, allowing a better understanding of ICI use in patients with CUP and potentially improving their efficacy [13].

The indications and extent of surgical resection are still controversial and their application is mostly limited. There is no standard for the timing, mode and scope of radiotherapy, but comprehensive treatment has been recognized by scholars [14]. The treatment of unknown primary metastatic cancer should be tailored to local conditions, different sites, different pathological types, different stages and available evidence regarding potential biomarkers in CUP. It has been recently reported that 28% of patients with CUP present one or more predictive biomarkers to immune checkpoint inhibitors(ICI), such as programmed death-ligand 1 (PD-L1) expression on ≥ 5% cancer cells in 22.5%(≥ 1% in 34%) and lymphocytes in 58.7%, microsatellite instability (MSI)-high in 1.8% and tumour mutational burden (TMB) ≥ 17 mutations per megabase in 11.8%. However, these biomarkers are not yet validated. Generally, CUP patients with TMB > 10 mutations per megabase have a trend for better outcomes when treated with ICI [15]. In this study, there were 7 patients who have similar features to known primary tumors. Therefore, the protocol was consistent with the primary tumor. Three female patients were treated with an ovarian cancer chemotherapy regimen due to clinical features similar to ovarian cancer, and two achieved complete response and one achieved partial response after treatment. Two male patients with metastatic adenocarcinoma of bone with elevated serum PSA were treated as metastatic prostate cancer, two patients both achieved complete response. The response to treatment for ovarian and prostate cancer has been very good, the reason why these five patients could achieve good results may be originated from the ovary and prostate. 2 patients were adenocarcinomas with immunohistochemical features of colorectal cancer which could be treated with chemotherapy according to the indications for metastatic colorectal cancer, they got partial response.19 patients received empirical treatment. The empirical chemotherapy regimen is carboplatin combined with epirubicin, etoposide. Patients treated empirically had a poor prognosis [16]. After completion of treatment,3 patients had PR, accounting for only 16% (3/19), another 4 patients were stable and 12 patients had disease progression.Empirical treatment did not show an advantage in this study. Gene expression profiling have also been exploited to determine a diagnosis for CUPs patients. 6patients with CUPs were predicted to have probable bowel cancer based on 64 gene expression profiles. After FOLFOX6 regimen chemotherapy, 3 cases had partial response, 1 case was stable and 2 cases progressed. Compared to imaging and histopathological methods, molecular molecular diagnosis has the advantages of high sensitivity and specificity, objective interpretation of results, etc. A large prospective non-randomised phase II study of 252 patients suggested that survival may be improved by site-specific therapy determined by a gene expression profile assay of the biopsy specimen, particularly for patients with a tissue of origin diagnosis of more responsive tumour types [17]. At the end of the follow-up, of the 32 patients, 2 were in complete remission, 3 were in partial remission, 4 were stable and 29 had progressive disease. Most people with CUPs did not respond well to treatment.

About the clinical diagnosis of CUPs, emphasis should be placed on the interplay of imaging, histopathology and molecular diagnostics to guide and corroborate each other. In the era of targeted therapies, accurate histopathological and molecular classification of tumours is essential, in order to administer the best tailored therapeutic strategy, the classifications based on epigenetic alterations have served this purpose. Indeed, cancer cells are characterized by a massive overall loss of DNA methylation(20–60% overall decrease in 5-methylcytosine), and by the simultaneous acquisition of specific patterns of hypermethylation at CpG islands of certain promoters, which can reversibly or irreversibly alter gene function, thereby contributing to cancer progression [18].

With advances in imaging, histopathology and molecular, the development of CUPs clinical diagnosis will be driven by the combination and complementarity of different tests with advances in imaging, histopathology and molecular diagnostic techniques. This will facilitate the development of clinical diagnosis of CUPs and help more patients to identify the primary site, and achieve precise treatment of cancer.

At the present time, there are significant deficiencies in the available studies comparing site-specific therapy and empiric chemotherapy. These deficiencies include patient accrual problems (oversampling treatment-resistant tumor types and long recruitment), study design limitations (observational and problematic trials), heterogeneity among the CUP classifiers(epigenetic vs. Transcriptomic profiling), and incomparable therapies. The assessment of recently published CUP literature allows to recommend two comprehensive clinical trial designs, a visionary and a pragmatic approach. Both are amenable to implementing the latest diagnostics and therapeutic advances to improve the quality of CUP research and the prognosis of many patients [19].

## Conclusions

The poorly differentiated nature of CUPs and lack of specific antigen detection, prevents primary tissue of origin diagnoses in these patients. Without the identification of a primary origin site, treatment is restricted to generic chemotherapy with limited benefit. In the future, with the development of genetic testing technology and the application of new targeted drugs, more and more patients with Cups will be better treated.

## Data Availability

The datasets used or analysed during the current study are available from the corresponding author on reasonable request.
